# Working Memory Performance During Concurrent Cycling at the First and Second Ventilatory Thresholds in Athletes

**DOI:** 10.3390/brainsci16101073

**Published:** 2026-10-06

**Authors:** Zafer Bilgin, Erdem Uylas, Muhammed Yerlikaya, Nurten Dinç, Erkan Günay, Murat Taş

**Affiliations:** 1 Department of Coaching Education, Faculty of Sport Sciences, Manisa Celal Bayar University, 45040 Manisa, Türkiye; zafer.bilgin@cbu.edu.tr (Z.B.); nurten.dinc@cbu.edu.tr (N.D.); murat.tas@cbu.edu.tr (M.T.); 2 Independent Researcher, 45040 Manisa, Türkiye; erdemuylas1995@gmail.com; 3 Institute of Postgraduate Studies, Department of Physical Education and Sports, Manisa Celal Bayar University, 45040 Manisa, Türkiye; mbyerlikaya612@gmail.com

**Keywords:** dual-task, working memory, ventilatory threshold, cognitive-motor interference, acute exercise, 2-back task

## Abstract

Background: How exercise intensity affects working memory during concurrent motor-cognitive tasks remains unclear. This study examined working memory during dual-task cycling at the first and second ventilatory thresholds (VT1, VT2). Methods: Eleven trained male endurance athletes (VO_2max_ 49.38 ± 5.69 mL/kg/min) completed a graded exercise test and two randomized crossover sessions of cycling at VT1 or VT2 with a concurrent 2-back task, with 2-back assessments before and after exercise. Hits, false alarms, reaction times and signal detection indices (*d′*, *c*) were analyzed using repeated-measures ANOVA and Holm-corrected planned contrasts. Results: At both intensities, hits and *d′* decreased during the dual-task (phase effects, *p* < 0.001), whereas the response criterion did not change. For hits, the primary outcome, the Condition × Phase interaction was significant [*F*(2, 20) = 4.90, *p* = 0.019, η^2^*p* = 0.329]: hits decreased substantially during the dual-task at VT2 (*d* = 1.21, *p*Holm = 0.010) but not significantly at VT1 (*p* = 0.074), and the VT1-VT2 difference during the dual-task was itself significant (*d* = 0.98, *p*Holm = 0.036). A planned contrast directly comparing the pre-to-dual-task change in hits between conditions supported this pattern (*p* = 0.022), although it did not survive the stricter Holm correction applied within that specific contrast family (*p*Holm = 0.089). Immediately after exercise, *d′* remained below pre-task values after VT2 (*p*Holm = 0.008) but not after VT1. Conclusions: Concurrent cycling at VT2 was associated with a significantly larger acute decline in working memory than cycling at VT1, directly supporting the study’s primary hypothesis. This intensity-dependent pattern, observed in a modest sample, should be confirmed in larger, adequately powered studies.

## 1. Introduction

Executive functions refer to higher-order cognitive control processes that enable the regulation of goal-directed behavior, the maintenance of attention, the selection of appropriate responses, and adaptation to changing environmental demands [1]. Among these processes, inhibition, working memory, and cognitive flexibility stand out as core components [2]. Working memory plays a critical role in the planning and regulation of both cognitive and motor behavior through the temporary maintenance, updating, and mental manipulation of information [3]. Accordingly, working memory performance is widely regarded as a central indicator for assessing executive function components such as attentional control, processing of task-relevant information, behavioral regulation, and appropriate response selection [4,5].

Dual-task conditions, in which motor and cognitive tasks are performed concurrently, are of particular importance for evaluating cognitive performance during exercise [6,7]. Under such conditions, individuals must sustain motor performance while simultaneously carrying out cognitive operations that require attention, information updating, and response selection [1]. While performing a motor action such as executing a sport-specific skill, an individual may also be required to retain information in mind, make decisions, or respond to external stimuli. This condition, referred to in the literature as the dual-task paradigm, generates neural competition between motor and cognitive processes due to the brain’s limited information-processing and attentional capacity [8]. Within the dual-task paradigm, because both tasks draw on shared central resources when performed concurrently, performance decrements may occur in one or both tasks a phenomenon defined as cognitive-motor interference [7]. In the present study, however, the motor component was deliberately held constant (fixed cadence and pedal load, actively maintained through verbal cues) rather than being allowed to vary and being measured as an outcome. Accordingly, the term cognitive-motor interference is used here in a restricted sense: it refers to the effect of exercise intensity on cognitive performance under a constant motor demand, rather than to bidirectional changes in both tasks. Within this framework, the intensity of the concurrently performed motor task or exercise may play a determining role in working memory performance, as one of the key factors shaping the allocation of cognitive resources. Exercise intensity is one of the most important determinants of the acute exercise-cognition relationship [9]. The effects of acute exercise on cognitive performance have been increasingly investigated in recent years. Current findings indicate that a single bout of exercise can produce beneficial effects on cognitive function, but that these effects vary depending on exercise intensity, duration, timing of administration, and the characteristics of the cognitive task employed [10,11]. Evidence for the facilitative effect of moderate-intensity aerobic exercise on cognitive performance is relatively robust, whereas the effects of high-intensity exercise appear more variable [9,11]. A study comparing moderate-intensity continuous exercise (MICE) with high-intensity interval exercise (HIIE) showed that 2-back reaction time was reduced immediately after both protocols in healthy young adults; at the 30-min post-exercise assessment, this improvement persisted after HIIE but had returned to the pre-exercise level after MICE. The same study suggested that this difference might be related to exercise-induced arousal, brain derived neurotrophic factor (BDNF) response, and changes in cerebral blood flow [12]. Because HIIE is intermittent and both protocols were assessed after, rather than during, exercise, these findings cannot be transferred directly to continuous exercise at fixed ventilatory thresholds; they nevertheless indicate that exercise intensity may shape the time course of working memory changes after exercise. Zheng et al. [13] reported that reaction time on an n-back task performed during moderate-intensity cycling exercise in young adults improved without a corresponding decline in accuracy, and that this improvement was accompanied by reduced oxygenated hemoglobin in prefrontal regions, including the dorsolateral prefrontal cortex, which is central to working memory, indicating that prefrontal working memory networks are modulated during moderate exercise.

Studies examining high-intensity exercise indicate that the effect on cognitive performance depends not only on exercise intensity but also on whether the cognitive task is administered during or after exercise. Sudo et al. [9] reported that cognitive performance tends to deteriorate more markedly during high-intensity exercise, under conditions in which physiological and cognitive demands increase simultaneously, whereas post-exercise findings are more heterogeneous. In contrast, Moreau and Chou [14] and Ai et al. [15] demonstrated that high-intensity exercise may produce small yet positive effects on executive functions, although this effect can vary according to the type of cognitive task, the timing of assessment, the exercise protocol, and participant characteristics. Moreira et al. [6], examining the dual-task paradigm in athletes, reported that acute dual-task exposure can lead to impairment in both motor and cognitive performance, a phenomenon that can be explained by the concept of “dual-task cost.” Consistently, Gutiérrez-Capote et al. [16] recently showed in basketball players that the executive demands and physical load of cognitive-motor dual-task interventions modulate both the acute and the chronic changes in inhibitory control, and that these effects depend on athletes’ baseline inhibitory capacity. Studies by Ji et al. [17] and Pellegrini-Laplagne et al. [18] in older adults, meanwhile, have shown that combined physical and cognitive exercise conditions can improve executive functions, although this improvement does not always surpass that achieved by single-domain physical or cognitive interventions. Taken together, these findings suggest that the exercise-cognition interaction varies according to intensity, task timing, task type, and dual-task load.

A review of the existing literature reveals that most studies have focused on cognitive tests administered at rest following acute exercise (post-exercise) [19]. Findings regarding how executive/working memory is affected during exercise particularly within a dual-task model incorporating different exercise intensities remain limited and inconsistent. Moreover, most existing studies define exercise intensity using relative percentages (%VO_2max_, %HR_max_) or subjective perception scales, which may overlook inter-individual physiological differences; the use of individualized ventilatory thresholds (VT1/VT2) has the potential to overcome this limitation. VT1 marks the first disproportionate increase in ventilation and CO_2_ output relative to O_2_ uptake and approximates the upper boundary of the moderate-intensity domain, whereas VT2, the respiratory compensation point, marks the onset of hyperventilation relative to CO_2_ output in response to developing metabolic acidosis and approximates the boundary of the severe-intensity domain [20,21]. Because the metabolic and ventilatory state associated with a given %VO_2max_ or %HR_max_ differs considerably between individuals, anchoring intensity to these thresholds ensures that all participants exercise in physiologically comparable domains, which is essential when asking whether cognitive performance differs between domains. Trained endurance athletes were studied because they are accustomed to sustained exercise at high metabolic load, and because their aerobic fitness allows sustained exercise at VT2 for the required duration. Based on the shared-resource account [7,8], we reasoned that the greater physiological demand at VT2 would leave fewer central resources available for the concurrent 2-back task than at VT1, predicting larger dual-task costs at VT2, and that residual effects of the heavier exercise bout would be reflected in lower performance in the immediate post-exercise assessment. Accordingly, the aim of the present study is to examine the acute effects of cognitive tests administered concurrently during motor tasks performed at different exercise intensities on individuals’ working memory performance. In this regard, we hypothesized that working memory performance would deteriorate more markedly at VT2 compared with VT1, and that this deterioration would still be evident in the immediate post-exercise assessment under the VT2 condition, rather than having returned to pre-task levels. These hypotheses were formulated a priori, before data collection, as part of the approved study protocol (Ethics Committee approval of 15 December 2023; grant no. 2024-061). The findings obtained are expected to provide important theoretical and practical contributions both to the design of training programs for the optimization of sport performance and to dual-task applications within neurocognitive rehabilitation processes.

## 2. Materials and Methods

### 2.1. Participants

The study included 11 male athletes aged 19.61 ± 2.14 years, with 9.15 ± 2.23 years of training experience, a height of 177.53 ± 10.28 centimeters (cm), a body weight of 66.10 ± 12.91 kilograms (kg), a body mass index (BMI, kg/m^2^) of 22.06 ± 2.92, and a maximal oxygen uptake (VO_2max_) of 49.38 ± 5.69 mL/kg/min. All participants were competitive, trained endurance athletes from cycling (*n* = 4), triathlon (*n* = 4) and athletics (*n* = 3) (VO_2max_ ≥ 40 mL/kg/min; see Section 2.7). All participants had prior experience with cycle ergometer exercise testing, having previously completed exercise tests in our laboratory using the same ergometer. Sample size was determined using G*Power 3.1.9.4 software (Heinrich Heine University, Düsseldorf, Germany). To determine an appropriate sample size for evaluating dual-task effects in athletes, the calculation was based on a within-subjects design for the primary outcomes of respiratory data (VO_2max_, VO_2_, VO_2_/HR, and VCO_2_) and cognitive task responses (hits, hit reaction time [RT], number of false alarms and false alarm RT), using an effect size (*f*) of 0.70, a significance level (*α*) of 0.05, and a statistical power (1–*β*) of 0.80. The anticipated effect size (f = 0.70) was not derived from a specific previous study; it was set a priori as a large effect, reflecting the expectation of substantial dual-task costs during exercise at VT2, and the calculation indicated a minimum required sample of 7 participants. Because the assumed effect size exceeds the conventional threshold for a large effect (f ≥ 0.40) [22], the sensitivity analysis reported below was used to characterize the effects that the study could realistically detect. The calculation used the “F tests—ANOVA: Repeated measures, within factors” procedure (a priori) and was powered to detect the main within-subject phase effect (pre-task vs. dual-task; one group, two measurements) for the primary outcomes, assuming a correlation among repeated measures of 0.50 and a nonsphericity correction of ε = 1 (noncentrality parameter λ = 13.72; critical *F*(1, 6) = 5.99; achieved power = 0.867). It did not address the Condition × Phase interaction or recovery-related contrasts, for which the study was not powered. However, this number was increased to 11 participants in order to enhance the statistical robustness of the data, account for variation in correlations among measurements, and allow for possible participant dropout. The participant group was composed of individuals who were not taking any medication and had no known mental or physical health conditions. Individuals who experienced a pathophysiological event during testing or who were using medication for psychological treatment were excluded from the study. A sensitivity analysis using the same G*Power procedure and assumptions (two measurements, correlation among repeated measures = 0.50, ε = 1, α = 0.05, power = 0.80, *n* = 11) indicated that the final sample could reliably detect only effects of f ≥ 0.47 (equivalent to a paired-samples dz ≈ 0.94); several condition-averaged and interaction effects fall below this threshold, and the findings should therefore be regarded as preliminary. No participant was excluded under the fitness (VO_2max_ ≥ 40 mL/kg/min) or other eligibility criteria, and all 11 athletes who were screened completed all four sessions without withdrawal.

### 2.2. Study Design

**Figure 1:**
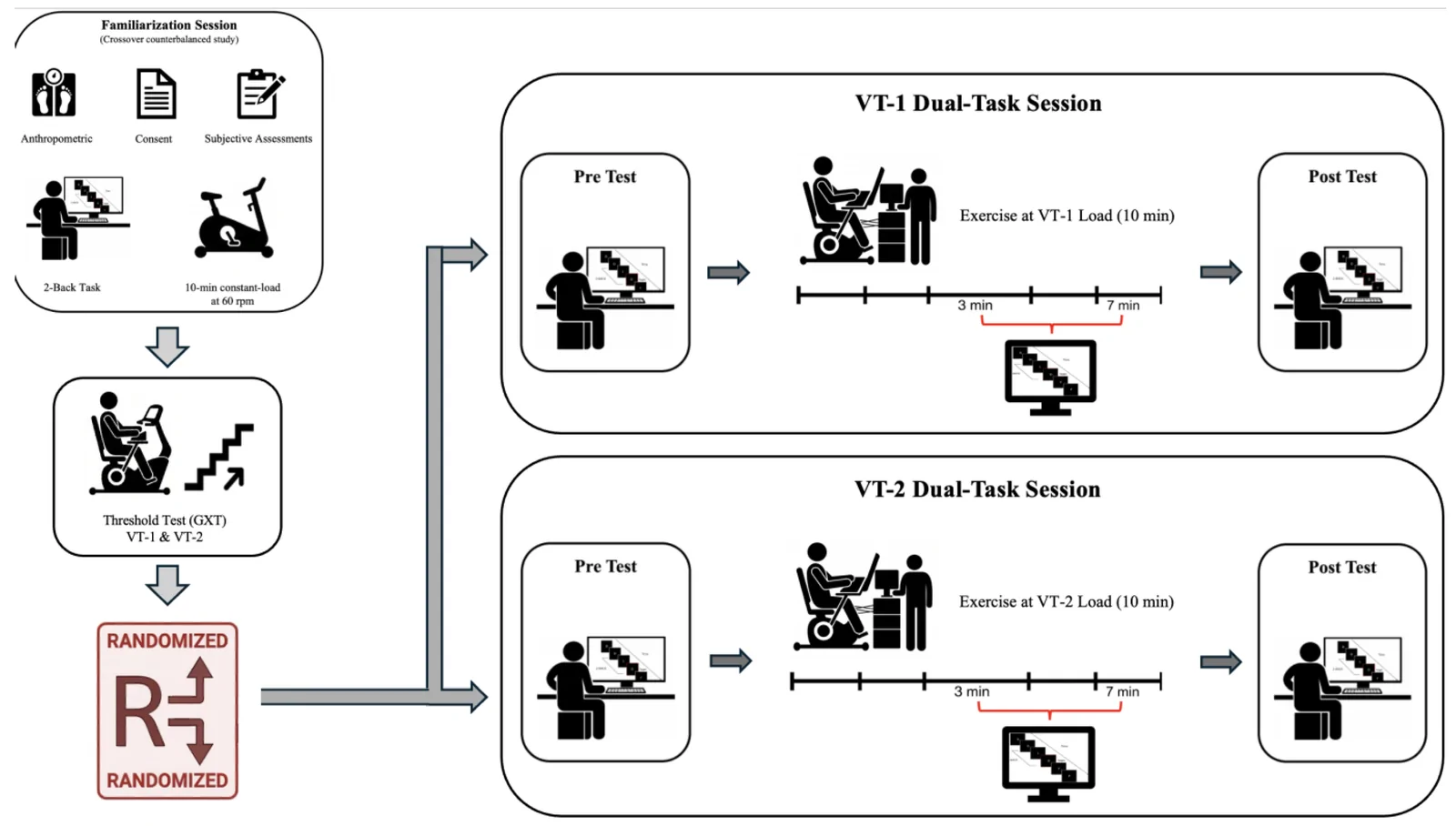
Overview of the four-session study design. GXT: graded exercise test; VT1/VT2: first/second ventilatory threshold.

The present study was conducted over a total of four sessions: familiarization, individual threshold determination, and dual-task sessions at the first and second ventilatory thresholds (VT1 and VT2). In the familiarization session, demographic and anthropometric data were collected after obtaining participants’ informed consent. This was followed by basic instruction and practice on the 2-back cognitive task. Participants were then familiarized with the cycle ergometer (Cosmed BK-24, COSMED, Rome, Italy) and adapted to the required cadence. In the subsequent session, participants who had been adapted to the cycle ergometer underwent a Graded Exercise Test (GXT) protocol (see Figure 1), following a 5-min warm-up, in which pedal load was increased by 20 watts per minute until volitional exhaustion. VO_2max_ values were determined from participants’ oxygen uptake responses at the point of exhaustion. In addition, the VT1 and VT2 thresholds and their corresponding pedal loads were calculated from gas exchange breakpoints. The GXT necessarily preceded the dual-task sessions, because the individual VT1 and VT2 workloads prescribed in those sessions had to be derived from it; only the order of the two dual-task sessions, which constitute the experimental comparison, was randomized. Because the GXT occupied the same position for all participants and was separated from the subsequent sessions by at least 48 h, any carry-over effect would be expected to affect both dual-task conditions equally. In the remaining two sessions, participants performed dual-task exercise at VT1 and VT2 pedal loads for 10 min each, in randomized order. In these sessions, a 2-back test was administered beforehand to determine baseline cognitive performance, followed by 10 min of exercise at the VT1 or VT2 load. The 2-back task began at the 3rd minute of exercise and continued until the 7th minute and was repeated at the end of exercise to determine the change in cognitive performance.

### 2.3. Familiarization Session

At the beginning of the session, the study protocol was explained to participants in detail, after which their written informed consent was obtained. Participants’ demographic information and anthropometric measurements were then collected. This was followed by instruction on the 2-back cognitive task, during which baseline performance values for this test were obtained [23]. To ensure participants’ adaptation to the cycle ergometer, a 10-min constant-load exercise bout was performed at 60 rpm [24].

### 2.4. Standardization

To exclude the effect of circadian rhythm, all test sessions were conducted at the same time of day (09:00–12:00). The laboratory environment was maintained at standard temperature (20–22 °C) and humidity (40–60%) levels [25]. Prior to all sessions, the Cosmed-Quark CPET gas analyzer (COSMED, Rome, Italy) was calibrated in accordance with the manufacturer’s instructions using ambient air and reference gas concentrations (16% O_2_, 5% CO_2_) [26]. Saddle height on the cycle ergometer was adjusted according to the length of each participant’s iliac bone, and handlebar distance was set according to an appropriate seating position; participants were seated in this fixed position, based on these reference values, in all sessions [25]. In addition, participants maintained a constant cadence (60 rpm) across all tests [24,27]. A fixed, relatively low cadence of 60 rpm was chosen because cadence itself influences the physiological response to cycling [24]; keeping it constant maintained comparable workload–VO_2_ relationships between the GXT and the dual-task sessions, and the low cadence also limited upper-body movement, facilitating stable keyboard responding. For the 2-back cognitive task, the test was repeated until a stable score was achieved (~2–3 rounds) in order to reduce learning effects [28]. Practice was continued until the researcher judged that performance no longer improved between consecutive rounds; no formal quantitative stability criterion was applied. Task comprehension was likewise verified by the researcher during the 1-back and 2-back practice blocks that preceded the main assessment. Participants also practiced responding on a keyboard integrated into the cycle ergometer while attempting to maintain a constant cadence (RPM) during performance of the 2-back task [29]. A standard USB mechanical keyboard was used for all response collection. Although commodity input devices can introduce non-trivial and variable timing latency in reaction-time research [30], the keyboard remained fixed in an identical position on the ergometer across all sessions and conditions (pre-task, dual-task, and post-task; VT1 and VT2). Any latency introduced by the hardware or by the cycling motion itself would therefore be expected to act as a constant offset across conditions and would not account for the condition and phase-dependent differences observed. A 48-h interval was maintained between sessions to prevent the accumulation of fatigue. In addition, participants were instructed to avoid alcohol and strenuous exercise for 24 h before testing, to abstain from caffeine and tobacco products for the final 12 h before testing, and to arrive at each session having fasted for at least 3 h and in a well-hydrated state, in line with standard pre-test recommendations for cardiopulmonary exercise testing [31]. Throughout the dual-task sessions, cadence (60 rpm) and pedal load were continuously monitored by the researchers; in the event of deviation, verbal cues were provided to participants during the intervals when the 2-back task was not being administered, in order to keep motor performance constant. Continuous monitoring confirmed that all participants maintained the prescribed cadence of 60 rpm during the 2-back block in both conditions; cadence was monitored visually on the ergometer display and was not recorded for quantitative analysis.

### 2.5. Anthropometric Measurements

Height was measured (to the nearest 0.01 cm) with participants barefoot using the height rod of the Tanita device; body weight and body fat percentage were measured using a Tanita bioelectrical impedance device (Tanita MC-780, Tanita Co., Tokyo, Japan) [32].

### 2.6. Body Mass Index (BMI)

Body mass index was calculated from height and body weight using the following formula [33]:

BMI = Weight (kg)/[Height (m)]^2^

### 2.7. Individual Threshold Determination Test (Graded Exercise Test—GXT)

At the beginning of this session, the gas analyzer (Cosmed-Quark CPET, COSMED, Rome, Italy) was connected to the participant, followed by a 1-min accommodation period at 60 rpm [26,34]. Participants then performed the GXT with incrementally increasing loads, during which respiratory parameters and thresholds were assessed using the Cosmed-Quark gas analyzer (COSMED, Rome, Italy) [26,34]. Participants first completed a 5-min warm-up (60 rpm) on the cycle ergometer (Cosmed BK-24, COSMED, Rome, Italy) [35]. This was followed by a protocol in which exercise load was increased incrementally (20 watts/min). Gas exchange was monitored continuously throughout the protocol, and heart rate responses were also recorded (SmartLab HRM-W) [36]. In particular, oxygen uptake (VO_2_), carbon dioxide production (VCO_2_), and ventilation (VE) were analyzed across the incremental loads. The validated V-slope method [37,38] was used to determine the first ventilatory threshold (VT1), second ventilatory threshold (VT2), and maximal oxygen uptake (VO_2max_) from the GXT data. This method takes into account the relationship between ventilation and load (watt) output. The V-slope method served as the reference method for determining VT1, with the point at which a disproportionate rise in VO_2_ was observed, together with the corresponding exercise load, defined as VT1. The point at which carbon dioxide output departed from linearity and began to rise disproportionately, together with the corresponding exercise load, was defined as VT2. As VT2 was operationally defined via the ventilatory/gas-exchange–based V-slope method (corresponding to the respiratory compensation point) rather than via blood lactate kinetics, blood lactate sampling was not performed to cross-validate this threshold; this is consistent with the ventilatory framework adopted throughout the study for both VT1 and VT2 determination. The maximal oxygen uptake and pedal load recorded between the 10th and 12th minutes, at the point of exhaustion, were defined as VO_2max_ [20]. Attainment of VO_2max_ was confirmed using secondary criteria in addition to volitional exhaustion: a respiratory exchange ratio (RER) ≥ 1.15 and a heart rate within 90–95% of the age-predicted maximum (220 − age). All participants met both secondary criteria. Throughout the VO_2max_ assessment, participants’ perceived exertion was measured using Borg’s Rating of Perceived Exertion scale (1970, 6–20 range) [39], and the test was terminated at the point at which participants reached exhaustion and could no longer continue. Participants with a VO_2max_ of 40 mL/kg/min or higher, corresponding to a moderate level of cardiorespiratory fitness, were included in the dual-task protocols [40,41]. The physical load thresholds to be used during the dual-task sessions were subsequently determined by two independent experts, blinded to participant data, using the V-slope method; in cases where the difference in inter-rater agreement exceeded 5%, a final value was determined by consensus with a third senior expert, and inter-rater reliability was assessed using the Intraclass Correlation Coefficient (a priori acceptance criterion: ICC ≥ 0.85) [21,42,43]. Inter-rater reliability, assessed with a two-way mixed-effects, single-measurement model [ICC(3,1)], met this criterion for both thresholds, both for consistency (VT1: ICC = 0.984; VT2: ICC = 0.999) and for absolute agreement (VT1: ICC = 0.978; VT2: ICC = 0.990).

### 2.8. Dual-Task Sessions

**Figure 2:**
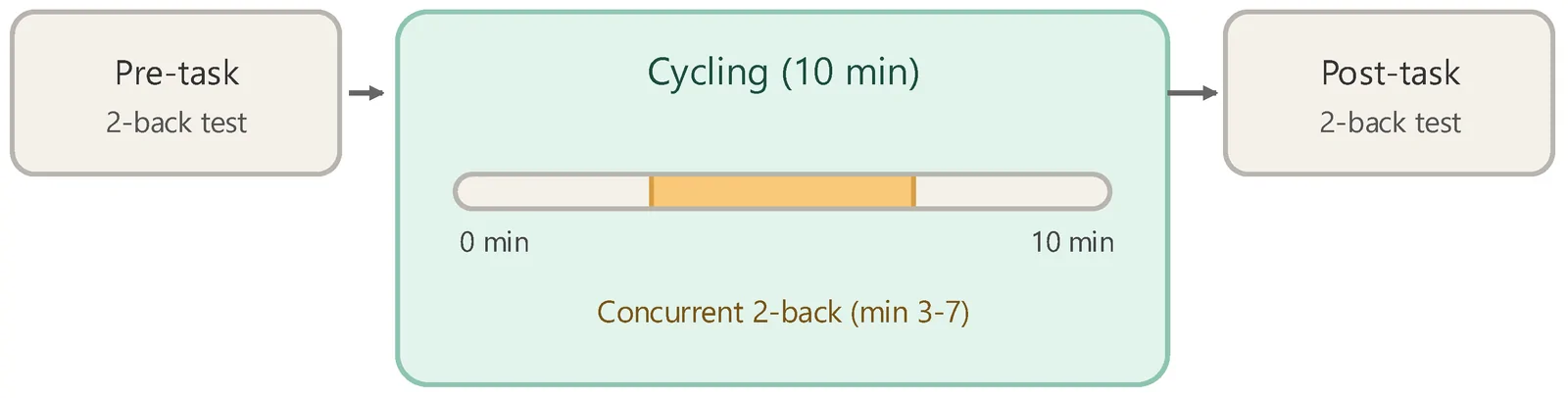
Within-session timeline of a dual-task trial. The 2-back task was performed concurrently with cycling between minutes 3 and 7.

In the dual-task sessions, participants’ physical and cognitive performance at different threshold loads (VT1 and VT2) was assessed [44]. This protocol was administered on separate days, following a randomized, crossover study design aimed at minimizing learning and fatigue effects. Cognitive performance was assessed with the 2-back test prior to each session (see Figure 2). Each dual-task session began with a brief period of unloaded, light pedaling at 60 rpm, following which the load was increased in a step transition to the target VT1 or VT2 intensity. Participants then pedaled the cycle ergometer at a constant cadence of 60 rpm while simultaneously completing the 2-back cognitive task presented on a screen positioned in front of them, with responses entered via a keyboard mounted on the ergometer at the VT1 and VT2 loads. The 2-back task began at the 3rd minute of exercise, once a steady state had been reached, and continued until the 7th minute. The 3-min lead-in was chosen because, after a step increase in work rate, the primary (fast) component of the VO_2_ response is normally completed within approximately 3 min [45], so that the cognitive task was performed under near-steady-state cardiorespiratory conditions; the 4-min window corresponded to the duration of the 2-back task. The total bout duration of 10 min was fixed a priori in the study protocol and was identical in both conditions; exercise was therefore continued from the 7th to the 10th minute after the 2-back block, so that the post-task assessment followed the same exercise duration at VT1 and VT2. A steady state was defined a priori as a change in VO_2_ of less than 150 mL/min across the 3rd-to-7th-minute window in which the dual-task was performed, an a priori threshold selected to represent a physiologically negligible change in oxygen uptake at this exercise intensity. This criterion was met in all participants at both VT1 and VT2, confirming that concurrent cognitive assessment occurred under stable cardiorespiratory conditions at both exercise intensities. The corresponding VO_2_ values are reported in Section 3.1. During this period, participants were instructed to maintain both their physical performance (cadence) and their performance on the 2-back cognitive task. At the end of exercise, the 2-back test was repeated immediately upon cessation of pedaling (within ~10–15 s) in order to determine the change in cognitive performance resulting from the dual-task load.

### 2.9. N-Back (2-Back) Task

The 2-back test was used to assess participants’ working memory function before and after the extended exercise session. This test measures the recognition of, and reaction time to, letters presented in a pseudo-randomized sequence on a computer screen, requiring identification of letters matching the one presented two positions earlier [46]. For the cognitive test, participants were seated at 70–90 cm from the computer screen, as recommended by Chu et al. [47]. All participants performed the test on the same computer, which featured a screen resolution of 2560 × 1664 and was an Apple Silicon model (Apple Inc., Cupertino, CA, USA; M2 processor, 13.6 inch). The test battery used was the 2-back task available in PEBL Launcher for PEBL, version 2.1 [28].

At the beginning of each session, participants first completed a 1-back task, followed by a 2-back task, to familiarize themselves with and practice the test. The researchers then administered the main test. In the main task, stimuli were presented to participants as a series of uppercase white letters (“C”, “H”, “K”, “N”, “R”, “W”, “X”, “Y”) displayed against a black background in the center of the computer screen. The inter-stimulus interval was 1500 ms, and the total number of stimuli was fixed at 120. The stimulus set consisted exclusively of consonants presented in a single uppercase font, avoiding vowels and pronounceable letter combinations, and the same letter set was used in all phases and conditions. When named in Turkish, however, several of these letters share a common vowel ending (e.g., “ce”, “he”, “ke”, “ne”, “re”, “ye”), which may increase phonological similarity; because this property was identical across all phases and conditions, it cannot account for the phase- or condition-dependent differences observed (see Section Limitations). Participants pressed the left shift key on the keyboard whenever the letter on screen matched the letter presented two positions earlier. Completion of the test took approximately 4 min. A new pseudo-randomized stimulus sequence was generated by the software for each administration (pre-task, dual-task and post-task), so that no participant was presented with the same sequence twice. Cognitive performance metrics were structured along two main dimensions: (1) the number of hits and false alarms, along with the corresponding reaction times, were recorded and analyzed; and (2) Signal Detection Theory (SDT) metrics, namely the sensitivity index and response criterion, were calculated—perceptual sensitivity index (*d′* = Z[Hit Rate] − Z[False Alarm Rate]) and response bias criterion (*c* = −0.5 × [Z (Hit Rate) + Z (False Alarm Rate)]) [48]. Hit and false-alarm rates were computed with the log-linear correction (0.5 added to the hit and false-alarm counts and 1 added to the numbers of target and non-target trials, i.e., 40 and 80) to avoid infinite z values; positive values of c indicate a conservative and negative values a liberal response bias.

### 2.10. Statistical Analysis

Statistical analyses were performed using JASP software (v0.16.2; University of Amsterdam, Amsterdam, The Netherlands) [49]. The normality of data distribution was verified using the Shapiro–Wilk test [50]. Paired-samples *t*-tests were used to compare cardiorespiratory parameters (relative VO_2_, absolute VO_2_, VO_2_/HR, and VCO_2_) between ventilatory thresholds (VT1 and VT2). The effects of exercise intensity and measurement time point on working memory and signal detection indices were examined using a 2 × 3 repeated-measures analysis of variance (RM-ANOVA), based on the factors Condition (VT1, VT2) and Phase (pre-task, dual-task, post-task). The dependent variables consisted of behavioral measures (number of hits, hit reaction time, number of false alarms, false alarm reaction time) and signal detection theory parameters [sensitivity index (*d′*) and response criterion (*c*)]. In cases where the assumption of sphericity was violated, the Greenhouse-Geisser correction was applied; significant main effects were further examined using Bonferroni-corrected post hoc tests. Planned pairwise comparisons (paired-samples *t*-tests) were conducted to examine acute within-threshold changes and recovery dynamics. The number of hits was defined as the primary outcome, because it most directly indexes successful working memory updating in the 2-back task; false alarms, reaction times and the SDT indices (*d′*, *c*) were secondary outcomes, and the correlation analyses were exploratory. In the interest of full transparency: the study’s original hypothesis, stated prior to data collection, concerned working-memory accuracy more broadly, and hits was the accuracy measure available without the additional assumptions required to compute *d′* and *c*; the explicit statistical designation of hits as the single primary outcome, and of *d′*, c, and the reaction-time/false-alarm measures as secondary, was articulated in this form during manuscript revision, in response to reviewer feedback, rather than fixed in a pre-registered analysis plan. We state this plainly rather than imply ex ante pre-specification of the full outcome hierarchy. A consolidated table of every planned contrast interpreted in this manuscript for the primary and secondary outcomes (hits, *d′*, and *c*), with sample size, test statistic, unadjusted *p* value, effect size, and Holm-adjusted *p* value, is provided in Section 3.4; the behavioral outcomes not driving any reported inference (hit reaction time, false alarms, false-alarm reaction time) are omitted from it for focus but are reported in full in the same section and were never significant at any contrast. For each outcome, the 2 × 3 RM-ANOVA constituted the omnibus test and distinguished a general phase effect (main effect of Phase), an overall condition difference (main effect of Condition), and a difference in change over time between conditions (Condition × Phase interaction). The planned contrasts corresponded to the hypotheses: (i) the pre-task to dual-task change within each condition; (ii) the direct comparison of this change between VT1 and VT2 (interaction contrast on change scores); and (iii) the pre-task to post-task difference within each condition and its comparison between conditions. Because planning contrasts in advance does not by itself control the family-wise error rate, Holm–Bonferroni correction was applied to the planned contrasts within families defined as the four behavioral outcomes (hits, hit RT, false alarms, false alarm RT) and the two SDT outcomes (*d′*, *c*) within each contrast; both unadjusted and Holm-adjusted *p* values (*p*Holm) are reported, and inferences are based on the adjusted values. Mean differences with 95% confidence intervals are reported for the central contrasts. Nonsignificant results were not interpreted as evidence of equivalence (e.g., preserved performance or complete recovery). Relationships between cardiorespiratory data and dual-task cognitive responses were determined using Pearson correlation analysis. These correlations were exploratory and not prespecified; 32 correlations were computed (4 cognitive × 4 cardiorespiratory parameters × 2 conditions), each based on all 11 participants, and because no multiplicity correction was applied for inference, they are interpreted descriptively. Effect sizes were reported as Cohen’s *d* for *t*-tests [22] and partial eta squared (*η^2^p*) for ANOVA [51]. For paired-samples *t*-tests, Cohen’s *d* was computed from the difference between the first and the second level of each comparison (e.g., pre-task − dual-task; VT1 − VT2), so that positive values indicate a decrease and negative values an increase from the first to the second level. For the Bonferroni-corrected post hoc comparisons of the RM-ANOVA, Cohen’s *d* values are those provided by JASP, which are based on the pooled standard deviation of the compared levels and are not corrected for multiple comparisons; they are therefore not directly comparable with the d values of the paired-samples *t*-tests. Analyses were based on all available data, with listwise exclusion within each analysis. Four raw response variables (hit count, hit reaction time, false-alarm count, false-alarm reaction time) for one participant’s VT2 dual-task session were initially absent from our analysis file because of a data-transcription gap, even though the session itself, its physiological recordings, and its PEBL-derived *d′*/*c* values were complete; the missing values were subsequently retrieved from the original source records and verified (13 hits + 27 misses = 40 targets, as expected) before being incorporated into the dataset used throughout this manuscript. Blank false-alarm counts, which occurred when a participant made no false alarms, were coded as zero, whereas the corresponding false-alarm reaction times remained missing. Consequently, *n* = 11 for hits, hit RT, false alarms, *d′*, and *c* in the RM-ANOVAs, and *n* = 8 for false-alarm RT, restricted to participants who registered at least one false alarm in every phase and condition; the n for each paired comparison is given by its degrees of freedom. Statistical significance was set at *p* < 0.05.

## 3. Results

### 3.1. Cardiorespiratory Responses and Physiological Intensity Verification

Cardiorespiratory parameters (relative VO_2_, absolute VO_2_, VO_2_/HR, and VCO_2_) at the first ventilatory threshold (VT1) and second ventilatory threshold (VT2) were compared using paired-samples *t*-tests. The results indicated significantly higher values at VT2 for relative VO_2_ [*t*(10) = −8.20, *p* < 0.001, *d* = −2.472], absolute VO_2_ [*t*(10) = −3.07, *p* = 0.012, *d* = −0.927], VCO_2_ [*t*(10) = −6.86, *p* < 0.001, *d* = −2.070], HR [*t*(10) = −4.83, *p* < 0.001, *d* = −1.46] and RER [*t*(10) = −4.93, *p* < 0.001, *d* = −1.49] (see Table 1); however, no significant change was observed in VO_2_/HR [*t*(10) = −0.54, *p* = 0.600, *d* = −0.163].

Mean RER values were below 1.0 at VT1 (0.984 ± 0.067) and above 1.0 at VT2 (1.184 ± 0.086). An RER above 1.0 indicates that CO_2_ output exceeded O_2_ uptake because of additional non-metabolic CO_2_ derived from bicarbonate buffering of accumulating hydrogen ions, which confirms that the VT2 workload was located above the transition to a substantial anaerobic energy contribution, in line with its operational definition as the respiratory compensation point. The a priori steady-state criterion (Section 2.8) was met by all participants; the mean change in VO_2_ between the 3rd and 7th minute of the dual-task was 90 ± 11 mL/min at VT1 and 115 ± 15 mL/min at VT2; all individual changes were below the 150 mL/min criterion.

### 3.2. Working Memory Performance Under Dual-Task Interference

Hits, hit reaction time, false alarms, and false alarm reaction time before and during the dual-task at the first ventilatory threshold (VT1) were compared using paired-samples *t*-tests (see Table 2; *n* = 11 throughout, following correction of a previously incomplete data point for one participant’s VT2 dual-task session; Section 2.10). None of the four outcomes changed significantly from pre-task to dual-task at VT1: hits [*t*(10) = 1.99, *p* = 0.074, *d* = 0.60], hit reaction time [*t*(10) = −1.54, *p* = 0.154, *d* = −0.46], false alarms [*t*(10) = −2.41, *p* = 0.037, *d* = −0.73; *p*Holm = 0.148, not significant after correction], or false alarm reaction time [*t*(10) = −0.72, *p* = 0.488, *d* = −0.22].

Hits, hit reaction time, false alarms, and false alarm reaction time before and during the dual-task at the second ventilatory threshold (VT2) were likewise compared (*n* = 11, except *n* = 8 for false alarm reaction time, restricted to participants who registered at least one false alarm in both phases). Hits decreased substantially [*t*(10) = 4.00, *p* = 0.0025, *d* = 1.21; *p*Holm = 0.010], while no significant changes were found for hit reaction time [*t*(10) = −1.24, *p* = 0.242, *d* = −0.37], false alarms [*t*(10) = −1.46, *p* = 0.174, *d* = −0.44], or false alarm reaction time [*t*(7) = −1.71, *p* = 0.132, *d* = −0.60].

Hits, hit reaction time, false alarms, and false alarm reaction time during the dual-task condition were compared between the VT1 and VT2 thresholds using paired-samples *t*-tests. Hits were significantly lower at VT2 than at VT1 during the dual-task condition [*t*(10) = 3.24, *p* = 0.009, *d* = 0.98]. However, no significant differences were found for hit reaction time [*t*(10) = −0.70, *p* = 0.502, *d* = −0.21], false alarms [*t*(10) = 0.86, *p* = 0.409, *d* = 0.26], or false alarm reaction time [*t*(10) = −0.07, *p* = 0.945, *d* = −0.02].

When signal detection theory (SDT) parameters were assessed using paired-samples *t*-tests, perceptual sensitivity (*d′*) showed a significant decrease from baseline during dual-task performance at VT1 [*t*(10) = 2.98, *p* = 0.014, *d* = 0.90], while this loss of sensitivity was considerably more pronounced at VT2 [*t*(10) = 6.15, *p* < 0.001, *d* = 1.85]. In contrast, the response criterion (*c*) did not change significantly from pre-task to dual-task at either VT1 [*t*(10) = 1.87, *p* = 0.091, *d* = 0.56] or VT2 [*t*(10) = −0.62, *p* = 0.547, *d* = −0.19]. When post-exercise recovery periods (post-task) were compared, *d′* scores during recovery following VT2 remained significantly lower than following VT1 [*t*(10) = 3.03, *p* = 0.013, *d* = 0.91], and the number of hits was also reduced [*t*(10) = 2.48, *p* = 0.033, *d* = 0.75]. Within-condition (pre vs. post) comparisons showed no significant difference in *d′* at VT1 between the pre-task and post-task assessments [*t*(10) = 1.29, *p* = 0.225, *d* = 0.39]; this nonsignificant difference indicates an absence of detectable residual change at this single timepoint and does not, by itself, establish that performance had returned to baseline. At VT2, by contrast, the loss of perceptual sensitivity remained significantly present during the post-task assessment [*t*(10) = 3.74, *p* = 0.004, *d* = 1.13]. This VT2 post-task reduction in *d′* survives Holm correction (*p*Holm = 0.008; Section 3.2), whereas the corresponding post-task reduction in hits does not (*p*Holm = 0.066); these two outcomes are therefore not treated as equally well-supported.

After Holm–Bonferroni correction, the decrease in hits from pre-task to dual-task at VT2 (*p*Holm = 0.010) and the VT1–VT2 difference in dual-task hits (*p*Holm = 0.036) remained significant, whereas the increase in false alarms from pre-task to dual-task at VT1 did not survive correction (*p*Holm = 0.148). The dual-task decreases in *d′* remained significant at both VT1 (*p*Holm = 0.028) and VT2 (*p*Holm < 0.001). Accordingly, the VT1-specific increase in false alarms should be interpreted with caution. For the pre-task versus post-task comparisons, the lower number of hits at VT2 did not survive correction (*p*Holm = 0.066), whereas the decrease in *d′* at VT2 remained significant (*p*Holm = 0.008).

The direct comparison of the pre-task to dual-task change in hits between conditions (interaction contrast on change scores; *n* = 11) indicated a larger decrease at VT2 than at VT1 (mean difference = 4.18 hits, 95% CI [0.73, 7.63]; *t*(10) = 2.70, *p* = 0.022), which did not remain significant after Holm–Bonferroni correction (*p*Holm = 0.089). The corresponding comparison of the pre-task to post-task change was not significant (mean difference = 2.36 hits, 95% CI [−0.82, 5.55]; *t*(10) = 1.65, *p* = 0.129). Within conditions, hits decreased from pre-task to dual-task by 2.09 (95% CI [−0.25, 4.43]) at VT1 and by 6.27 (95% CI [2.78, 9.76]) at VT2, and were lower in the post-task than in the pre-task assessment by 0.27 (95% CI [−1.90, 2.44]) at VT1 and by 2.64 (95% CI [0.59, 4.68]) at VT2.

### 3.3. Hit Rate Across Exercise Intensities and Time Course

A two-way repeated-measures ANOVA examining the effects of time (pre-task, dual-task, and post-task) and ventilatory threshold (VT1 and VT2) on the number of hits revealed significant main effects of both time [*F*(2, 20) = 11.62, *p* < 0.001, *η^2^p* = 0.537] and threshold condition [*F*(1, 10) = 7.34, *p* = 0.022, *η^2^p* = 0.423] on hit counts. Critically, the Condition × Time interaction was also significant [*F*(2, 20) = 4.90, *p* = 0.019, *η^2^p* = 0.329], indicating that the pattern of change in hits across phases genuinely differed between VT1 and VT2. This analysis included the full sample of 11 participants, following correction of a previously incomplete VT2 dual-task data point for one participant (Section 2.10).

Because the interaction was significant, simple effects are reported in preference to the main-effect post hoc comparisons alone. As detailed in Section 3.2, hits did not change significantly from pre-task to dual-task at VT1 [*t*(10) = 1.99, *p* = 0.074], whereas at VT2 the decrease was substantial and significant [*t*(10) = 4.00, *p* = 0.0025, *p*Holm = 0.010]. The VT1-VT2 difference during the dual-task itself was significant [*t*(10) = 3.24, *p* = 0.0089, *p*Holm = 0.036]. A planned contrast directly testing the interaction—the pre-to-dual-task change in hits, compared between conditions—supported this pattern [mean difference = 4.18 hits, 95% CI (0.73, 7.63); *t*(10) = 2.70, *p* = 0.022], although it did not survive the stricter Holm correction applied within the four-outcome interaction-contrast family (*p*Holm = 0.089). Taken together, the omnibus interaction, the condition-specific simple effects, and the directional pattern of the planned contrast converge on the same conclusion: dual-task performance declined more steeply at VT2 than at VT1.

For descriptive completeness, Bonferroni-corrected post hoc comparisons for the main effect of phase (collapsed across condition) indicated that hits were lower during the dual-task phase than the pre-task phase (*p*bonf = 0.010, *d* = 1.16) and recovered during the post-task phase relative to the dual-task phase (*p*bonf = 0.033, *d* = −0.94); the pre-task versus post-task difference did not survive correction (*p*bonf = 0.122, *d* = 0.71). The main effect of condition (collapsed across phase) also favored VT1 over VT2 [*t*(10) = 2.71, *p* = 0.022, *d* = 0.82]. As with any significant interaction, these condition- and phase-collapsed averages describe the overall pattern but are secondary to the simple effects reported above for interpreting the condition-specific trajectory. Descriptively (Table 2), hits decreased from 20.73 ± 2.87 (pre-task) to 18.64 ± 3.50 (dual-task) at VT1 and from 21.55 ± 2.54 to 15.27 ± 6.07 at VT2, and were 20.45 ± 3.86 and 18.91 ± 3.59, respectively, in the post-task assessment; the larger standard deviation during the VT2 dual-task indicates considerable inter-individual variability in the magnitude of this response.

### 3.4. Processing Speed and Error Frequency

None of the main effects of phase or condition, nor the condition × phase interaction, reached statistical significance for hit reaction time, number of false alarms, or false alarm reaction time (*p* > 0.08; *n* = 11 throughout, except *n* = 8 for false alarm reaction time, restricted to participants who registered at least one false alarm in every phase and condition). Mean and standard deviation values for all reaction time and error parameters are presented in Table 2. Descriptive statistics for the signal detection indices are presented in Table 3, complete RM-ANOVA results for all outcomes in Table 4, and all planned contrasts interpreted for hits, *d′*, and *c* in Table 5.

### 3.5. Signal Detection Parameters: Perceptual Sensitivity (d′) and Response Criterion (c)

A two-way repeated-measures ANOVA on the sensitivity index (*d′*) revealed a significant main effect of phase [*F*(2, 20) = 22.02, *p* < 0.001, *η^2^p* = 0.688]. Neither the main effect of condition [*F*(1, 10) = 1.80, *p* = 0.209, *η^2^p* = 0.153] nor the Condition × Phase interaction [*F*(2, 20) = 1.59, *p* = 0.228, *η^2^p* = 0.137] was significant, indicating that although hits showed an intensity-dependent pattern (Section 3.3), the composite sensitivity index declined similarly in both conditions. Bonferroni post hoc comparisons for the main effect of phase (*n* = 11) revealed that sensitivity declined significantly during the dual-task relative to pre-task [*t*(10) = 6.77, *p*bonf < 0.001, *d* = 2.04], and although it increased again during the post-task recovery window [*t*(10) = −3.04, *p*bonf = 0.037, *d* = −0.92], it remained significantly below the initial resting baseline [*t*(10) = 3.50, *p*bonf = 0.017, *d* = 1.06].

Evaluation of the response criterion (*c*) revealed no significant main effect of phase [*F*(2, 20) = 0.86, *p* = 0.439, *η^2^p* = 0.079]. The main effect of condition approached but did not reach significance [*F*(1, 10) = 4.66, *p* = 0.056, *η^2^p* = 0.318], and the Condition × Phase interaction was not significant [*F*(2, 20) = 2.27, *p* = 0.130, *η^2^p* = 0.185]. Participants adopted a conservative criterion (*c* > 0) in all phases and conditions (Table 3); this criterion showed a trend toward differing between VT1 and VT2 but did not change significantly during or after the dual-task within either condition.

### 3.6. Cardiopulmonary Correlates of Cognitive Control During Dual-Task Execution

These analyses were exploratory of the 32 correlations computed (*n* = 11 at VT1 and at VT2 for all pairs except relative VO_2_-false alarm RT, which remains *n* = 10 pending body-mass data required to compute relative VO_2_ for the corrected participant), three reached *p* < 0.05 without correction, and none remained significant after Holm–Bonferroni correction; the full correlation matrix is provided in Supplementary Table S1. Relationships between dual-task response parameters (hits, hit reaction time, false alarms and false alarm reaction time) and cardiorespiratory parameters (relative VO_2_, absolute VO_2_, VO_2_/HR, and VCO_2_) were examined using Pearson correlation analysis. A positive association that was significant only without correction for multiple comparisons was found between relative VO_2_ and false alarm reaction time during the VT2 dual-task condition (*r* = 0.661, *p* = 0.037).

No significant association was found between absolute VO_2_ and false alarm reaction time during the VT1 dual-task condition (*r* = −0.545, *p* = 0.083). Likewise, during the VT2 dual-task condition, no significant associations were found between absolute VO_2_ and both the number of hits (*r* = 0.598, *p* = 0.052) and hit reaction time (*r* = −0.586, *p* = 0.058).

No significant association was found between oxygen pulse (VO_2_/HR) and false alarm reaction time during the VT1 dual-task condition (*r* = −0.600, *p* = 0.051). During the VT2 dual-task condition, a negative association that was significant only without correction for multiple comparisons was found between VO_2_/HR and hit reaction time (*r* = −0.622, *p* = 0.041).

No significant association was found between carbon dioxide output (VCO_2_) and hit reaction time during the VT1 dual-task condition (*r* = −0.552, *p* = 0.078). During the VT2 dual-task condition, a moderate, negative association that was significant only without correction for multiple comparisons was found between VCO_2_ and hit reaction time (*r* = −0.664, *p* = 0.026).

## 4. Discussion

The present study aimed to examine the effects of a concurrently administered 2-back cognitive test, performed during acute motor tasks at different ventilatory thresholds (VT1 and VT2), on athletes’ working memory performance. The main finding was that concurrent cycling was associated with reduced working memory performance at both intensities, and that this decline was significantly larger at VT2 than at VT1, directly supporting the study’s primary hypothesis: the Condition × Phase interaction for hits, the primary outcome, was statistically significant [*F*(2, 20) = 4.90, *p* = 0.019, η^2^*p* = 0.329], and this pattern was corroborated by significant condition-specific simple effects and a planned interaction contrast (Section 3.3). At VT1, the number of hits did not decrease significantly, while a nominally significant increase was observed in the number of false alarms, which did not survive correction for multiple comparisons (*p* = 0.037, *d* = −0.726); at VT2, by contrast, a marked and highly significant decrease in hits (*p* = 0.0025, *d* = 1.21, *p*Holm = 0.010) was observed, and the VT1-VT2 difference during the dual-task was itself significant (*p*Holm = 0.036; Section 3.3). This pattern indicates that, as exercise intensity increases, cognitive-motor interference (CMI) differs not only in magnitude but also in kind; the significant Condition × Phase interaction, together with the condition-specific simple effects, provides statistical support for a genuine difference between conditions, rather than a merely descriptive one. Given that exercise intensity in the literature is typically defined using relative percentages (%VO_2max_, %HR_max_) or subjective perception scales [9,11], and that cognitive tests are most often administered after exercise [19], the present study offers a distinct contribution: by using ventilatory thresholds (VT1/VT2) individualized according to athletes’ own physiological profiles, it tested cognitive-motor interference in real time within a concurrent dual-task paradigm, with findings demonstrating that a significantly more pronounced cognitive decline is associated with the second ventilatory threshold (VT2), supported by a significant Condition × Phase interaction for hits. Furthermore, pre-task/post-task comparisons conducted within this same individualized threshold framework showed that, in the immediate post-exercise assessment, hits did not differ significantly from pre-task values after VT1, whereas they remained lower after VT2, suggesting that exercise intensity may influence not only performance during exercise but also performance immediately afterwards. This dual finding (concurrent impairment plus condition-dependent differences in immediate post-exercise performance) is notable in that it unifies the “during-exercise” and “post-exercise” cognitive-effect paradigms often examined separately in the literature within a single individualized threshold framework. This approach both increases individual physiological precision in the measurement of exercise intensity and renders the findings more directly translatable to training practice, such as determining the intensity and recovery duration of dual-task training. In interpreting these findings, we draw on several theoretical frameworks from the cognitive and exercise sciences; these are not presented as competing explanations but as complementary perspectives that may each capture a different facet of the observed cognitive-motor interference.

In interpreting these results, several types of evidence converge on a consistent picture for hits, the primary outcome, while diverging for the secondary signal-detection indices. First, the RM-ANOVA showed a general phase effect across all three cognitive outcomes: hits, *d′*, and *c* all changed significantly or near-significantly across phases for at least one measure, with hits and *d′* declining during the dual-task, indicating that concurrent cycling was associated with poorer working memory performance at both intensities. Second, there was a significant overall condition difference in hits, averaged across phases. Third, and most importantly, the Condition × Phase interaction was significant for hits [*F*(2, 20) = 4.90, *p* = 0.019], indicating that the time-course of change genuinely differed between VT1 and VT2; this was corroborated by the condition-specific simple effects (significant decline at VT2, not at VT1) and by a planned interaction contrast on change scores (Section 3.3). The interaction did not reach significance for *d′* (*p* = 0.228) or c (*p* = 0.130), meaning that for these composite signal-detection indices the present data do not provide statistical evidence that the pattern of change differed between conditions, even though the within-condition simple effects for *d′* were themselves more pronounced at VT2 than at VT1 (see below). The VT1-VT2 difference in hits discussed below is therefore treated as a statistically supported condition effect, while the parallel discussion of *d′* is treated more cautiously, as a descriptive pattern consistent with, but not independently confirmed by, the hits finding; the mechanistic accounts that follow remain interpretive, as the observed cognitive changes do not directly establish resource depletion, central regulation, or hypofrontality as mechanisms.

To make the contribution of each framework explicit, their distinctive predictions for the present paradigm can be summarized as follows. The limited-capacity model [52] predicts that dual-task costs scale with total combined demand and should therefore increase monotonically from VT1 to VT2 across performance indices. Multiple-resource theory [53] additionally predicts that interference depends on the structural overlap between tasks; because rhythmic lower-limb cycling and a visually presented, manually answered 2-back task share relatively few specific resources, it predicts small costs at moderate intensity, with larger costs emerging only when the general (central) demand rises. The Central Governor Model [54] makes a regulatory prediction: as homeostatic threat increases, the motor task should be protected at the expense of the secondary cognitive task, so that cognitive decrements should track markers of systemic physiological strain (e.g., VCO_2_, ventilation) rather than task difficulty per se. The Transient Hypofrontality Hypothesis [55] predicts a selective impairment of prefrontal-dependent processes (here, working memory updating indexed by hits and *d′*) at high intensity, with rapid restoration once exercise ceases. Finally, the inverted-U model predicts facilitation at moderate intensity and impairment only at high intensity. Against these predictions, the present data show no facilitation at VT1 (partial support for the inverted-U model), a larger decline in hits and *d′* at VT2 (consistent with the limited-capacity, multiple-resource and hypofrontality accounts), a single significant association between VCO_2_ and hit reaction time at VT2 (weakly consistent with the Central Governor Model), and incomplete post-exercise recovery at VT2 (not predicted by a purely transient hypofrontality account). Because the present design did not manipulate structural task overlap, did not measure prefrontal activity, and did not quantify motor output as an outcome, these predictions could not be discriminated directly; the frameworks are therefore used as interpretive rather than confirmatory accounts. Importantly, because the design included neither an exercise-only nor a cognitive-task-only control condition (see Section Limitations), none of the interpretations below can fully separate dual-task resource competition from a more general effect of exercise-induced physiological strain (e.g., breathlessness, discomfort, or attentional capture by interoceptive signals) on cognition; this caveat applies equally to the Central Governor and hypofrontality accounts developed below.

Several theoretical frameworks offer possible, non-exclusive explanations for this pattern; the present design cannot adjudicate between them; and we present them accordingly, as interpretive possibilities rather than confirmed mechanisms. Kahneman’s limited-capacity model and the multiple-resource theories that followed it [52,53] attribute dual-task costs to competition for shared central resources. The qualitatively different pattern at VT1 (preserved hits, alongside a nominal increase in false alarms that did not survive correction for multiple comparisons) versus VT2 (a significant decline in hits and a numerically larger, though not independently significant, reduction in *d′*; Section 3.3) is broadly compatible with this account; “resource depletion” is used here only as an interpretive label, not a directly measured construct. Noakes’s Central Governor Model [54] offers an alternative, central-regulation account in which systemic physiological strain approaching VT2 could prioritize motor output over cognitive processing. This is compatible with, but not established by, the exploratory association between VCO_2_ and hit reaction time (*r* = −0.664, *p* = 0.026, uncorrected for multiplicity), and, as noted in Section Limitations, the present design cannot separate this account from a more general effect of exercise-induced physiological strain on cognition in the absence of a matched resting control condition. The stable response criterion (*c*) across phases and conditions (Table 3) suggests the dual-task decrement reflected reduced discrimination ability rather than a shift in response strategy [56]. Finally, the behavioral pattern observed at VT2—a marked decline in both hits and *d′*—is also consistent with Dietrich’s Transient Hypofrontality Hypothesis [55], though without neuroimaging data we cannot test this directly; future studies combining fNIRS or EEG with this paradigm [13,57] could help distinguish between these accounts.

Our findings partially align with the classic inverted-U model of the relationship between exercise intensity and cognitive performance: moderate-intensity exercise (around VT1) may facilitate or at least preserve cognitive performance, whereas high-intensity exercise (at or above VT2) may impair it. Our data provide statistical support for a threshold-like pattern around VT2 rather than a simple linear relationship: the Condition × Time interaction for hits was significant [*F*(2, 20) = 4.90, *p*= 0.019], and the magnitude of the VT2-specific decline, together with the significant simple effect at VT2 and the near-significant interaction contrast on change scores (Section 3.3), is consistent with a more abrupt, rather than purely gradual, decline in performance as exercise intensity crosses this threshold. Because only two intensities were tested, however, the present design cannot determine the shape of the function between VT1 and VT2 or beyond VT2; the possibility that the respiratory compensation point represents a genuine cognitive breakpoint, as opposed to one point on a steeper but still continuous dose–response curve, remains a hypothesis for future studies testing several intensities below and above VT2. Indeed, Sudo et al. [9] reported that cognitive performance tends to deteriorate more markedly under conditions in which cognitive and physiological loads are simultaneously high, a finding consistent with the marked performance decline observed under the VT2 dual-task condition in our study. Pre-task and post-task comparisons suggested an asymmetry between the VT1 and VT2 conditions in the immediate post-exercise assessment, which began approximately 10–15 s after exercise cessation and lasted about 4 min. In the VT1 condition, no significant difference was found between pre-task and post-task hits (*p* = 0.785, *d* = 0.084); this nonsignificant difference, however, does not demonstrate complete recovery. In contrast, in the VT2 condition, a significant difference between pre-task and post-task hits was found (*p* = 0.017, *d* = 0.867), indicating that performance at VT2 remained below pre-task levels during the immediate post-exercise assessment, although this difference did not survive Holm–Bonferroni correction (*p*Holm = 0.066), whereas the corresponding decrease in *d′* did (*p*Holm = 0.008), consistent with the broader pattern of a significantly larger and more persistent disruption at VT2 established above. Because only a single post-exercise assessment was performed, these data do not describe a recovery trajectory or indicate how long any residual effect persists. This finding diverges somewhat from a fully transient hypofrontality picture, as proposed by Dietrich [55], and suggests that the metabolic/cardiopulmonary recovery process around VT2 that is, the rate at which VO_2_ and VCO_2_ levels return to resting values may also be associated with post-exercise cognitive performance, a possibility that requires repeated post-exercise assessments to test.

### Limitations

Despite these valuable findings, this study has several limitations that should be addressed in future research. First, the sample size was limited to 11 participants; the sensitivity analysis (Section 2.1) indicates that the study was adequately powered only for large within-condition effects (dz ≥ 0.94), so smaller effects than those observed here could not have been reliably detected. While the Condition × Phase interaction for hits, the primary outcome, did reach significance despite this modest sample, the corresponding interactions for the secondary indices *d′* and *c* did not, and the non-significance of those specific interactions may still reflect insufficient power rather than a true absence of a condition-specific effect for those measures; this asymmetry across outcomes, and the overall sample size, mean the present findings should be replicated in larger, adequately powered samples before the magnitude of the effect is considered established. In addition, the group consisted exclusively of male athletes; despite active recruitment efforts, no eligible female endurance athletes volunteered within the recruitment window of the study, so the sample was not restricted to men by design. This restricts the generalizability of the findings to female athletes or to different sedentary populations. Second, although the keyboard-based 2-back task performed at a constant cadence on the cycle ergometer provided high internal validity within the laboratory setting, it does not fully capture the dynamic and unpredictable motor-cognitive demands encountered in field settings (external validity). Additionally, the response latency of the ergometer-mounted keyboard (Section 2.4) was not independently benchmarked; future studies could quantify it by comparing keyboard response latency during active pedaling with a stationary baseline. Moreover, because the motor component was held constant by design, motor performance was not quantified as an outcome, and potential bidirectional interference could not be assessed. The letters used in the 2-back task share a common vowel ending when named in Turkish, which may have increased phonological similarity, although identically across all conditions. In addition, dietary intake before testing was neither standardized nor recorded beyond the minimum 3-h fasting period. Third, the present design compared cognitive performance at rest (pre-task) against performance during concurrent cycling (dual-task), without an exercise-only control condition in which participants exercised at the same intensities without performing the cognitive task. Consequently, the observed decline in working memory performance cannot be fully attributed to dual-task resource competition alone; it may also partly reflect a direct effect of physiological strain (e.g., breathlessness, discomfort, or attentional capture by interoceptive signals) on cognitive performance, independent of the concurrent-task demand itself. An exercise-only condition would allow motor costs to be quantified and would show whether prior concurrent cognitive activity alters post-exercise performance, but it would not by itself isolate the mechanism of cognitive impairment during exercise. Likewise, no time-matched cognitive-task-only control condition (seated, without exercise) was included. Although the pre-task and post-task assessments were performed without exercise, they were not matched in time or number of task repetitions to the dual-task block; therefore, part of the dual-task and post-task changes could reflect repeated administration of the 2-back task within the session (e.g., practice effects or mental fatigue) rather than cycling per se. A matched resting condition with repeated cognitive testing at the same time points would help distinguish exercise-related changes from time and repeated-testing effects. Future studies employing larger and more heterogeneous samples and directly monitoring cerebral cortical activation during exercise using methods such as fNIRS or EEG, would provide a clearer account of the neural origins of this cognitive-motor bottleneck. Furthermore, because only two exercise intensities were tested, the shape of the intensity–performance relationship (gradual versus threshold-like) could not be determined, and because only a single immediate post-exercise assessment was performed, the time course of cognitive recovery could not be characterized. Finally, the correlation analyses were exploratory and underpowered and should not be taken as evidence for a physiological mechanism. With *n* = 11, a correlation of |r| ≥ 0.60 is required to reach *p* < 0.05, and both the correlation coefficients and the effect sizes reported here are therefore imprecise and sensitive to individual data points.

## 5. Conclusions

Concurrent cycling at both VT1 and VT2 was associated with a decline in working memory performance, with hits and perceptual sensitivity decreasing during the dual-task and partially returning toward pre-task values after exercise cessation. Critically, the primary hypothesis that this decline would be larger at VT2 than at VT1 was supported: the Condition × Phase interaction for hits, the primary outcome, was statistically significant [*F*(2, 20) = 4.90, *p* = 0.019], and this pattern was corroborated by significant condition-specific simple effects (a significant decline at VT2 but not at VT1) and a directionally consistent planned interaction contrast. At VT1, hits did not decrease significantly and the increase in false alarms did not survive correction for multiple comparisons, whereas at VT2 hits decreased markedly and significantly, even after Holm correction. These findings indicate that cognitive-motor interference intensifies as the respiratory compensation point is approached, a conclusion that should be extended in future, adequately powered studies incorporating several exercise intensities. Moreover, in the immediate post-exercise assessment, hits did not differ significantly from pre-task values after VT1 but remained lower after VT2 (significant for *d′* after correction for multiple comparisons, and nominally so for hits); because only a single post-exercise assessment was performed, this neither demonstrates complete recovery at VT1 nor characterizes the full duration of recovery at VT2.

These findings are directly relevant to managing cognitive load during exercise: working memory performance is reduced during exercise at both moderate and high intensity, and this cost is significantly larger at or above VT2 than at VT1. This has practical implications both for training and competition planning in sports requiring tactical decision-making, and for neurocognitive rehabilitation programs in which dual-task applications are structured progressively: referencing individualized ventilatory thresholds (particularly VT2) when calibrating the intensity of dual-task training is supported by the present findings as a means of managing both performance and safety, although confirmation in larger, adequately powered samples remains warranted given the modest size of the present sample (*n* = 11). Our results offer a timely contribution to the exercise-cognition literature regarding how “during-exercise” cognitive performance can be linked to individualized physiological thresholds.

## Figures and Tables

**Table 1:** Cardiorespiratory Parameters. Values are mean ± SD. VT1 vs. VT2 (paired-samples *t*-tests): relative VO_2_, *p* < 0.001; absolute VO_2_, *p* = 0.012; VCO_2_, *p* < 0.001; heart rate, *p* < 0.001; oxygen pulse, *p* = 0.600; RER, *p* < 0.001.

Ventilation Thresholds	Relative VO_2_ (mL/kg/min)	Absolute VO_2_ (mL/min)	VCO_2_ (mL/min)	Heart Rate (Bpm)	Oxygen Pulse (O_2_/HR)	RER VCO_2_/VO_2_
VT1	35.88 ± 3.94	2814 ± 389.8	2854.87 ± 414.33	155.98 ± 14.70	18.08 ± 3.63	0.984 ± 0.067
VT2	44.14 ± 5.74	3190 ± 438.1	3789.03 ± 459.95	173.96 ± 11.04	18.43 ± 3.13	1.184 ± 0.086

**Table 2:** 2-Back Cognitive Test results.

Cognitive Task Response	Baseline	VT1/VT2	Pre-Task	Dual-Task	Post-Task
Hit	21.54 ± 3.61	VT1	20.73 ± 2.87	18.64 ± 3.50	20.45 ± 3.86
		VT2	21.55 ± 2.54	15.27 ± 6.07	18.91 ± 3.59
Hit Reaction Time (msec)	539.03 ± 105.94	VT1	535.33 ± 88.80	557.59 ± 84.82	548.08 ± 112.52
		VT2	535.15 ± 93.51	589.39 ± 183.31	548.73 ± 124.71
False Alarm	5.82 ± 6.82	VT1	3.73 ± 3.66	7.27 ± 6.36	6.27 ± 8.51
		VT2	3.82 ± 3.95	5.64 ± 4.32	6.55 ± 7.84
False Alarm Reaction Time (msec)	642.29 ± 138.39	VT1	591.17 ± 122.43	623.66 ± 129.81	590.87 ± 241.89
		VT2	551.54 ± 87.37	628.02 ± 205.19	606.34 ± 134.27

**Table 3:** Signal detection indices across conditions and phases.

Index	Condition	Pre-Task	Dual-Task	Post-Task
*d′*	VT1	1.78 ± 0.51	1.32 ± 0.56	1.61 ± 0.73
	VT2	1.89 ± 0.63	1.19 ± 0.60	1.43 ± 0.61
*c*	VT1	0.85 ± 0.15	0.75 ± 0.19	0.78 ± 0.20
	VT2	0.85 ± 0.22	0.92 ± 0.25	0.78 ± 0.18

Values are mean ± SD (*n* = 11 for all cells, following correction of a previously missing VT2 dual-task data point for one participant; see Section 2.10). *d′*: perceptual sensitivity index; c: response criterion (positive values indicate a conservative response bias). Both indices were computed with the log-linear correction (see Section 2.9).

**Table 4:** Repeated-measures ANOVA results for all cognitive outcomes.

Outcome	*n*	Effect	df	*F*	*p*	η^2^*p*
Hits	11	Condition	1, 10	7.34	0.022	0.423
		Phase	2, 20	11.62	<0.001	0.537
		Condition × Phase	2, 20	4.90	0.019	0.329
Hit RT	11	Condition	1, 10	0.34	0.575	0.033
		Phase	2, 20	2.20	0.137	0.180
		Condition × Phase	2, 20	0.45	0.647	0.043
False Alarms	11	Condition	1, 10	0.67	0.432	0.063
		Phase	2, 20	2.76	0.088	0.216
		Condition × Phase	2, 20	0.62	0.550	0.058
False Alarm RT	8	Condition	1, 7	0.00	0.978	0.000
		Phase	2, 14	1.99	0.174	0.221
		Condition × Phase	2, 14	0.85	0.450	0.108
*d′*	11	Condition	1, 10	1.80	0.209	0.153
		Phase	2, 20	22.02	<0.001	0.688
		Condition × Phase	2, 20	1.59	0.228	0.137
*c*	11	Condition	1, 10	4.66	0.056	0.318
		Phase	2, 20	0.86	0.439	0.079
		Condition × Phase	2, 20	2.27	0.130	0.185

Condition: VT1 vs. VT2; Phase: pre-task, dual-task, post-task. Greenhouse-Geisser-corrected degrees of freedom are reported for effects involving Phase. *n*: participants with complete data for the respective outcome (listwise exclusion; see Section 2.10).

**Table 5:** Consolidated planned contrasts for the primary outcome (hits) and the secondary signal-detection outcomes (*d′*, *c*), added in response to reviewer feedback requesting a single transparent summary of every interpreted contrast, its sample size, test statistic, unadjusted *p* value, effect size (Cohen’s *d*), and Holm-adjusted *p* value (*p*Holm). Asterisks and bold *p*Holm values indicate contrasts that survive Holm–Bonferroni correction within their family (behavioral outcomes: hits, hit RT, false alarms, false alarm RT, m = 4; SDT outcomes: *d′* and *c*, m = 2; interaction contrasts on change scores form their own respective families). The three behavioral outcomes not shown here (hit RT, false alarms, false alarm RT) did not yield a significant contrast at any comparison and are reported in full in Table 4.

Outcome	Contrast	*n*	t (df)	Raw *p*	d	*p*Holm
**Hits**	VT1 pre-dual	11	1.99 (10)	0.074	0.60	0.222
	VT2 pre-dual	11	4.00 (10)	0.003	1.21	0.010 *
	dual VT1 vs. VT2	11	3.24 (10)	0.009	0.98	0.036 *
	post VT1 vs. VT2	11	2.48 (10)	0.033	0.75	0.130
	VT1 pre-post	11	0.28 (10)	0.785	0.08	1.000
	VT2 pre-post	11	2.87 (10)	0.017	0.87	0.066
***d′***	VT1 pre-dual	11	2.98 (10)	0.014	0.90	0.028 *
	VT2 pre-dual	11	6.15 (10)	<0.001	1.85	<0.001 *
	dual VT1 vs. VT2	11	1.36 (10)	0.204	0.41	0.204
	post VT1 vs. VT2	11	3.03 (10)	0.013	0.91	0.025 *
	VT1 pre-post	11	1.29 (10)	0.225	0.39	0.293
	VT2 pre-post	11	3.74 (10)	0.004	1.13	0.008 *
***c***	VT1 pre-dual	11	1.87 (10)	0.091	0.56	0.091
	VT2 pre-dual	11	−0.62 (10)	0.546	−0.19	0.546
	dual VT1 vs. VT2	11	−2.06 (10)	0.066	−0.62	0.132
	post VT1 vs. VT2	11	−0.17 (10)	0.870	−0.05	0.870
	VT1 pre-post	11	1.57 (10)	0.147	0.47	0.293
	VT2 pre-post	11	1.41 (10)	0.189	0.43	0.189
**Hits**	Interaction contrast (Δ pre → dual, VT2 vs. VT1)	11	2.70 (10)	0.022	0.81	0.089
***d′***	Interaction contrast (Δ pre → dual, VT2 vs. VT1)	11	1.18 (10)	0.264	0.36	0.394
***c***	Interaction contrast (Δ pre → dual, VT2 vs. VT1)	11	−1.38 (10)	0.197	−0.42	0.394

## Data Availability

The data presented in this study are available on request from the corresponding author. The data are not publicly available due to privacy restrictions, as the small sample size (*n* = 11) combined with individual physiological and demographic variables could allow participant identification.

## Supplementary Materials

The following supporting information can be downloaded at: https://www.mdpi.com/article/10.3390/brainsci16101073/s1. Table S1: Pearson correlations between cardiorespiratory parameters and dual-task cognitive responses at the first (VT1) and second (VT2) ventilatory thresholds (exploratory analysis; 32 correlations).
