# Supplementary material for: Working Memory Performance During Concurrent Cycling at the First and Second Ventilatory Thresholds in Athletes
Source: Brain Sci. 2026 Oct 6;16(10):1073. doi: 10.3390/brainsci16101073 (PMC13702500; doi:10.3390/brainsci16101073)
Supplement: Supplementary file 1 [file brainsci-16-01073-s001.zip › brainsci-4615533-supplementary.pdf]

Supplementary Material

Table S1. Pearson correlations between cardiorespiratory parameters and dual-task cognitive responses at the first (VT1) and second (VT2) ventilatory thresholds (exploratory analysis; 32 correlations).

Condition	Cardiorespiratory	Cognitive (dual-task)	n	r	p	p (Holm)
VT1	Relative VO_2	Hits	11	-0.286	0.393	1.000
VT1	Relative VO_2	Hit RT	11	0.165	0.628	1.000
VT1	Relative VO_2	False alarms	11	0.443	0.173	1.000
VT1	Relative VO_2	False alarm RT	11	-0.051	0.882	1.000
VT1	Absolute VO_2	Hits	11	0.031	0.928	1.000
VT1	Absolute VO_2	Hit RT	11	-0.590	0.056	1.000
VT1	Absolute VO_2	False alarms	11	-0.082	0.810	1.000
VT1	Absolute VO_2	False alarm RT	11	-0.545	0.083	1.000
VT1	VO_2/HR	Hits	11	0.251	0.457	1.000
VT1	VO_2/HR	Hit RT	11	-0.418	0.201	1.000
VT1	VO_2/HR	False alarms	11	-0.141	0.679	1.000
VT1	VO_2/HR	False alarm RT	11	-0.600	0.051	1.000
VT1	VCO_2	Hits	11	-0.193	0.570	1.000
VT1	VCO_2	Hit RT	11	-0.552	0.078	1.000
VT1	VCO_2	False alarms	11	0.016	0.962	1.000
VT1	VCO_2	False alarm RT	11	-0.489	0.127	1.000
VT2	Relative VO_2	Hits	10	-0.053	0.884	1.000
VT2	Relative VO_2	Hit RT	10	0.293	0.412	1.000
VT2	Relative VO_2	False alarms	10	0.170	0.639	1.000
VT2	Relative VO_2	False alarm RT	10	0.661	0.037	1.000
VT2	Absolute VO_2	Hits	10	0.607	0.062	1.000
VT2	Absolute VO_2	Hit RT	10	-0.589	0.073	1.000
VT2	Absolute VO_2	False alarms	10	-0.213	0.555	1.000
VT2	Absolute VO_2	False alarm RT	10	-0.150	0.679	1.000
VT2	VO_2/HR	Hits	10	0.513	0.129	1.000
VT2	VO_2/HR	Hit RT	10	-0.606	0.063	1.000
VT2	VO_2/HR	False alarms	10	-0.191	0.597	1.000
VT2	VO_2/HR	False alarm RT	10	-0.090	0.804	1.000
VT2	VCO_2	Hits	10	0.246	0.493	1.000
VT2	VCO_2	Hit RT	10	-0.650	0.042	1.000
VT2	VCO_2	False alarms	10	-0.364	0.301	1.000
VT2	VCO_2	False alarm RT	10	-0.414	0.234	1.000

Relative VO_2: mL/kg/min; absolute VO_2 and VCO_2: mL/min; VO_2/HR: oxygen pulse; RT: reaction time. p (Holm): Holm–Bonferroni-adjusted across all 32 correlations. At VT2, dual-task data were available for 10 participants.
